# 

**Published:** 1893-11-11

**Authors:** 


**OUR NEW OFFICES,**
428, Strand, W.C., will henceforth be the Office of this Journal.

**Notices of Births, Deaths, and Marriages** are inserted in this column at a charge of 2s. 6d. for an announcement not exceeding four lines.

### NOTICE TO CORRESPONDENTS.

All MS., Letters, Books for Review, and other matters intended for the Editor should be addressed The Editor, The Lodge, Porchester Square, London, W.

The Editor cannot undertake to return rejected MS., even when accompanied by stamped directed envelope.

**Notice to Advertisers and Subscribers.**

All Advertisements, Orders for Copies of The Hospital, and Business Communications should be addressed to The Publisher, *not to the Editor*, The Hospital, 428, Strand, W.C.

The Cost of Subscription, post free, is as follows:—Per week, 2½d.; per quarter, 2s. 6d.; per half-year, 5s.; per annum, 8s. 6d Foreign:—Per Annum, 10s. 8d.; payable, in all cases, in advance.

**"Burdett's Hospital Annual," 1893.**

Fourth year of publication. 700 pp. Boards, gilt lettering. A most complete guide to British, Colonial, Indian, and American Hospitals, Dispensaries, Nursing and Convalescent Institutions, and Asylums. Price 5s. Published by The Scientific Press (Limited), 428, Strand, W.C.

**NOTICE.—The Index for Vol. XIV. (ending Sept. 30th, 1893) is on sale at the Office of this Journal, price 2½d., post free.**

### SCRAPS AND GLEANINGS.

The Forfar Infirmary has just completed a most satisfactory year during which 123 in-patients have undergone treatment.

The Committee of the Alton Hospital Sunday Fund have decided to hold their collections in future on Saturdays instead of on Sundays.

Considerable sanitary improvements have been made in the Montrose Infirmary, and the drainage has now been rendered generally efficient.

A convalescent home is to be provided for patients from the Cork hospitals by the generous gift of Sir John Arnott. Negotiations have been opened for the purchase of a site at Youghal.

A concert was recently given at Belfast in aid of the Hospital for Consumption in that town. The building has been recently enlarged and accommodation for ten more in-patients provided.

During the past quarter 181 cases of scarlatina have been received at the Gorse Hill Infectious Diseases Hospital, Swindon. The hospital is now full and extra isolation accommodation is required.

The annual church parade of the London Colney Friendly Societies in aid of the St. Alban's Hospital and Dispensary was recently held. The offertories at the services and the money collected in the streets amounted to £20.

A bazaar has been held at Gravesend in aid of the children's ward of the Gravesend Hospital, a leading part in the proceedings being taken by children. Nearly £300 was realised, and a Children's Hospital Union was formed.

Ambulance classes will shortly be commenced at Shrewsbury. There will be five classes, including one for nursing. Last year the pupils numbered 150, but it is thought that this number will be considerably increased this year.

We are glad to hear that the response to the appeal issued on behalf of the Ottery St. Mary Cottage Hospital is sufficient to warrant the continuance of the work as commenced by Mrs. Elliot, though increased support is still much needed.

The Hereford Board of Guardians have provided an infectious diseases hospital for the use of their indoor, outdoor, and casual paupers. The building, which is now completed, is of corrugated iron, and affords accommodation for seven persons.

The attendance of members of the Friendly Societies of Devizes at their annual hospital Sunday parade, was not so good as in former years. The sum of £35 which was collected in the churches and streets was devoted to the Devizes Cottage Hospital.

During the past quarter, 144 indoor and 590 outdoor patients have been received at the Bedford General Infirmary. New beds of the Lawson Tait type have been provided, and the building has been repainted. The institution is in urgent need of increased support.

### Books Received.

Horace Cox.

"Tales in Verse." By J. A. Goodchild.

Bailliere, Tindall, and Co.

"Cancer and Its Complications." By Charles E. Jennings, M.D.

Century Company, New York,

"Characteristics." By S. Weir Mitchell, M.D.

**Periodicals and Pamphlets.** — London: The Humanitarian, "Action of Alcohol on the Human Body" by W. Valentine Bird, M.D. Cassell's Family Magazine, Reich's Medicinal Anzeiger, The Psalms of David and Modern Criticism, Child's Companion, The Cottager and Artizan, Friendly Greeting, The Leisure Hour, The Sunday at Home, The Girl's Own Paper, The Boy's Own Paper.

### Medical Vacancies and Appointments.

### APPOINTMENTS.

W. D. Arnison, M.D., B.S., M.R.C.S., L.R.C.P., appointed Anæsthetist to the Royal Infirmary, Newcastle-on-Tyne. T. Beattie, M.B., B.S., appointed Senior House Physician to the Royal Infirmary, Newcastle-on-Tyne. George Blacker Elliott, L.R.C.S.I., L.M.L.A.H.Dub., appointed Medical Officer of Health to the Brixham Local Board. A. Ellison, M.R.C.S., L.R.C.P., appointed one of the House Surgeons to the General Infirmary, Leeds. E. G. Firth, L.S.A., appointed Resident Medical Officer to the Ida Hospital of the General Infirmary, Leeds. E. L. Fox, M.A., M.D.Camb., M.R.C.P.Lond., appointed Physician to the South Devon and East Cornwall Hospital, Plymouth. Arthur George Haydon, M.R.C.S.Eng., L.R.C.P. Lond., appointed Assistant Electrician to St. Bartholomew's Hospital. E. Hofton, M.R.C.S., L.R.C.P., appointed Resident Obstetric Officer to the General Infirmary, Leeds. D. N. Jackson, M.B., B.S., appointed House Surgeon to the Royal Infirmary, Newcastle-on-Tyne. Henry W. Jacob, M.A., M.D. Dub., appointed Honorary Physician to the Taunton and Somerset Hospital. Robert Jardine, M.D.Edin., M.R.C.S.Eng., appointed Physician for Diseases of Women and Children to the Public Dispensary, Glasgow, and Lecturer on Midwifery and Gynæcology to the Lady Missionary Training Home, Westercraig, Glasgow. Benjamin Evans Jones, L.R.C.P. and S.Edin., L.F.P.S.Glasg., appointed Lecturer to the County Council Technical Instruction Committee (Kirkham and district centre) on Sick Nursing and Ambulance for the Winter Session 1893 94. David Keele, M.R.C.S., L.R.C.P., appointed Honorary Surgeon to the Holloway and North Islington Dispensary. Frederick H. Lewis, B.A., M.B., B.C., L.R.C.P., M.R.C.S., appointed House Surgeon to the Royal Alexandra Hospital for Sick Children, Brighton. Albert Morton Martin, M.B.Durh., B.S., appointed Surgical Registrar to the Royal Infirmary, Newcastle-on-Tyne. C. Oldfield, M.R.C.S., L.R.C.P., appointed one of the House Surgeons to the General Infirmary, Leeds. Robert Robert, M.B., C.M.Edin., appointed Professor of Anatomy in the University of Durham. H. Smurthwaite, M.B., B.S., appointed Junior House Physician to the Royal Infirmary, Newcastle-on-Tyne. W. Percy Stocks, F.R.C.S., appointed Assistant Surgeon to the Clinical Hospital, Manchester. W. W. Stoney, M.B.Vict., appointed one of the House Physicians to the General Infirmary, Leeds. George Herbert Williams, M.D., M.R.C.S.Eng., L.R.C.P.Edin., appointed Medical Examiner to the United States Branch of the Nederland Life Insurance Company (Limited).

### NOTES FROM THE SCHOOLS.

Westminster Hospital Annual Students' Dinner takes place at the Criterion, Piccadilly, on November 16th, at 7.30 p.m. Dr. Bourns will occupy the chair.

Westminster Hospital Rugby Football Club were successful against Windsor on Saturday at the call of "no side." The scores were Westminster Hospital, 8 points; Windsor, 0.

### VACANCIES.

**Resident Medical Officers.**

Cheltenham General Hospital. Resident Surgeon for the Branch Dispensary; unmarried, or if married, without family. Salary £180 per annum, with partly-furnished house, coal, and gas. Applications to the Honorary Secretary and Treasurer by November 25th.

Kent and Canterbury Hospital. Assistant House Surgeon; unmarried. Salary £50 per annum, with board and lodging. Applications to the Secretary by November 30th.

London Throat Hospital, 204, Great Portland Street, W. Junior Assistant Surgeon. Applications to Edward Law, M.D., Honorary Secretary, Medical Committee, by November 14th.

National Sanatorium for Consumption and Diseases of the Chest, Bournemouth. Resident Medical Officer and Secretary. Salary £120 per annum, with board and lodging. Applications to the "Chairman of Committee" by December 5th.

Oldham Infirmary. House Surgeon; doubly qualified. Salary £50 per annum, wi h board and lodging. Applications to the Secretary by November 14th.

Royal Free Hospital, Gray's Inn Road, W.C. Junior Resident Medical Officers, two (House Physicians); doubly qualified. Board, residence, and washing only. Applications to C. W. Thies, Secretary, by November 27th. No canvassing of Board permitted.

Royal National Hospital for Consumption, Ventnor. Assistant Resident Medical Officer; unmarried. Salary £70 per annum, with board and lodging in the hospital. Applications to be addressed to the Board of Management at the London office, 34, Craven Street, Charing Cross, W.C.

Salford Union Infirmary. Assistant Medical Officer; doubly qualified. Salary £130 per annum, with furnished apartments. Applications, endorsed "Assistant Medical Officer," to T. H. Bagshaw, Clerk to the Guardians, Union Offices, Eccles New Road, Salford, by November 14th.

Staffordshire General Infirmary. House Surgeon; doubly qualified. Salary £100 per annum, with board, lodging, and washing. Applications to the Secretary of the Infirmary, Lloyd's Bank, Limited, Stafford, by November 18th.

Sunderland Infirmary. House Physician; doubly qualified. Salary £80 per annum, rising £10 annually to £100, with board and residence. Applications to the Chairman of the Medical Board by December 4th.

Surrey Dispensary, Great Dover Street, S.E. House Surgeon; doubly qualified; unmarried, and over 30 years of age. Salary £120 per annum, with furnished apartments, coal, gas, and attendance. Candidates must attend before the Monthly Committee on Tuesday, November 14th, at six p.m.

Torbay Hospital and Provident Dispensary, Torquay. Junior House Surgeon and Dispenser; doubly qualified, unmarried. Board, lodging, and attendance provided. Salary £80 per annum. Applications to the Honorary Secretary, Captain Phillpotts, R.N., by Nov. 13th.

### Non-Resident Medical Officers.

Ballyshannon Union (Kinlough Dispensary). Medical Officer. Salary £100 per annum, with £20 yearly as Medical Officer of Health, together with registration and vaccination fees. Applications to Mr. James McGurran, Honorary Secretary. Election on November 23rd.

Hospital for Women, Soho Square, W. Assistant Physician. Applications to the Secretary by November 14th.

### THE EDINBURGH TRIPLE QUALIFICATION.

### Papers Recently Set.

FIRST EXAMINATION.

Questions in Elementary Biology (five questions, of which four only are to be answered. Illustrate your answers by means of sketches).

1. Give an account of living protoplasm, specifying its chief physiological characteristics.
2. Describe the nervous system in a lobster. Where are the auditory organs lodged?
3. Mention the bones in the fore-limb (wing) of a pigeon. To which are the "secondary" quills attached?
4. Detail the minute features of *Bacterium termo*, and say what a "zooglœa" is.
5. Describe fully the inflorescence of the rose, and the structure of the "receptacle."

### PASS LISTS.

### University of London.

PRELIMINARY SCIENTIFIC (M.B.) EXAMINATION: JULY, 1893.

Entire Examination.

First Division.—J. T. Auld, Owens College; H. W. Bruce, Guy's Hospital; J. F. Dobson, Yorkshire College; C. H. Fagge, Guy's Hospital; R. C. Field; Yorkshire College; Mildred M. Gostling, Bedford College, London; Bessie N. Hamilton, University College, Dundee; Alice M. Hawker, University College; R. Kelsall, Owens College; J. Mooney, Owens College; J. Moreton, Owens College; S. Pearce, B.A., private study and University Tutorial College; G. E. Richmond, B.A., Owens College; J. H. Sheldon, Owens College; S. M. Smith, Epsom College; Mabel G. Stevenson, University College; W. H. M. Telling, Guy's Hospital; C. J. Thomas, University College, Cardiff; F. E. Walker, Guy's Hospital; P. M. Wright, Kingswood School.

Second Division.—E. W. Adams, Firth College; K. B. Alexander, Guy's Hospital; J. Broadley, Yorkshire College; C. W. Chaplin St. Olave's Grammar School, Southwark; H. H. Cheesman, Westminster Hospital; G. H. Coltart, Epsom College; S. W. R. Colyer, Charing Cross Hospital; H. Davies, University College, Aberystwith; W. A. Dewhurst, Yorkshire College; H. D. Everington, St. Bartholomew's Hospital; Lucinda C. E. Forster, Bedford College, London; E. V. Foss, University College, Bristol; E. A. Gates, University College and private study; J. A. Glover, St. Paul's School; F. G. Greenwood, Yorkshire College; H. FitzN. Hine, Middlesex Hospital and University Tutorial College; W. J. Hirst, Yorkshire College; H. H. Jamieson, St George's Hospital and St. Paul's School; H. S. Jenkins, Craigmore College, Bristol; A. J. Knowlton, Hartley Institute and Royal College of Science; Ethilda B. M. Meakin, University College; W. T. Milton, Guy's Hospital; E. W. Mole, B.A., private study; Edith Nield, School of Medicine, Edinburgh, and Heriot-Watt College; R. Norman, Epsom College; J. B. Page, St. Mary's Hospital; H. B. Palmer, Edinburgh School of Medicine and private study; P. A. Ross, University College, Bristol; W. T. Rowe, St. Bartholomew's Hospital; P. W. Rowland, St. Bartholomew's Hospital; C. Salkeld B.A., Durham College of Medicine and Science, Newcastle-on-Tyne; H. H. Scott, St. Bartholomew's Hospital and City of London School; A. P. H. Simpson, University College, Liverpool, and private study; F. B. Skerrett, Newcastle-under-Lyme High School and Owens College; A. J. Stanley, Mason College; E. Taunton, private tuition and City Polytechnic; A. Tedman, University College, Bristol; W. H. Unwin, University and Cheshunt Colleges; R. F. C. Ward, Epsom College; Eliza T. Watts, private study; G. E. Waugh, Epsom College, C. A. West, Westminster Training University, Tuitorial, and City of London College; R. W. Woodward, B.A., University College, Nottingham, and private study.

Honours Candidate Recommended for a Pass.—J. A. N. Longley, King Edward High School, Birmingham, and Mason College.

Chemistry and Experimental Physics.—H. R. Beale, St. Thomas's

Hospital and private tuition; J. W. H. Bendle, St. Mary's Hospital; T. P. Berry, Guy's Hospital; S. O. Bingham, St. Thomas's Hospital; H. Bond, St. Bartholomew's Hospital; Susila A. Bonnerjee, private study; F. Brickwell, St. Bartholomew's Hospital; J. D. Bridger, Guy's Hospital and private study; J. C. Briscoe, King's College; Josephine Brown, private tuition; Janet W. Carr, University Tutorial College; H. F. Cheshire, private study; W. H. Coltart, Mason College; A. G. Colvile, University Tutorial College; B. N. Das, University College and Hughli College, Bengal; F. S. Dawe, University Tutorial College; E. J. Distin, King's College; R. H. Dixon, private tuition; E. P. H. Dudley, Firth College; H. Dyer, Epsom College; C. E. Evans, Bourne College, Quinton, and University Tutorial College; H. A. T. Fairbank, Epsom College; E. L. Farncombe, Malvern and Mason Colleges; J. S. Gayner, University Tutorial College and St. Bartholomew's Hospital; H. B. Gibbins, Leighton Park School, University Tutorial College and private study; M. H. Greener, private study; L. E. C. Handson, Guy's Hospital and private study; A. C. Haslam, St. Thomas's Hospital; R. Hatfield, St. Bartholomew's Hospital; E. A. Hodder, Bedford Grammar School; J. G. F. Hosken, St. Bartholomew's Hospital; J. H. Howgate, private study and Yorkshire College; E. T. Jensen, Guy's Hospital; F. H. Lawson, Middlesex Hospital and University Tutorial College; Frances A. Leete, private study and Birkbeck Institution; F. C. Lewis, St. Mary's Hospital and Birkbeck Institution; J. M. A. Manning, St. George's Hospital and Birkbeck Institution; G. C. Marrack, St. Bartholomew's Hospital; J. Matthews, Firth College; T. Morgan, University College, Aberystwith; A. Mort, Royal College of Science, Dublin; J. R. Morton, London Hospital; B. N. Mullan, Grant College, Bombay, University Tutorial College, and private study; J. S. New, private study; H. G. B. Newham, St. Thomas's Hospital and Birkbeck Institution; A. P. Newton, Mason College and private study; J. A. O'Dowd, Mason College; Major B. Oliver, University College and private study; A. Orme, private study; C. Pike, London Hospital; B. E. Potter, Birkbeck Institution and Charing Cross Hospital; W. B. Prowse, Clifton Laboratory and private study; Louise Rickword, Bedford College, London; J. E. Robinson, St. Bartholomew's Hospital; S. A. Razzak, Guy's Hospital; F. W. Sheppard, University Tutorial College, G. B. Thwaites, University Tutorial College; and J. F. West, King Edward School, Birmingham, and Mason College.

Biology.—E. G. Andrew, Guy's Hospital; D. A. Ashton, Owens College; Constance Mary Bourlay, Mason College and private study; Lucy Bramley-Moore, Bedford College, London; F. Butterfield, Owens College; H. Calvert, University College; E. T. Caton, B.A., University Extension College, Reading, and private study; W. H. Cazaly, B.A., St. Bartholomew's Hospital; F. G. H. Cooke, University College; F. W. Cotton, University College, Bristol; D. Davies, University College; E. F. Ellis, University College; E. Evans, University College, Bangor; T. Evans, University College, Aberystwith; C. B. Fairbank, Epsom and University College and private study; T. L. Fennell, Owens College; B. G. Fiddian, University College, Cardiff; E. F. Fookes, Owens College; H. E. C. Fox, Guy's Hospital; F. A. Hadley, King's College; Helen Beatrice Hanson, Westcombe House, Brighton; W. C. Hill, University Tutorial College; B. F. Hussey, St. Mary's Hospital; O. Inchley, University College, Nottingham; F. A. Johns, University Tutorial College; N. H. Joy, St. Bartholomew's Hospital; H. C. F. Keall, University College, Bristol; E. Leach, Owens College; R. C. Leaning, Kent College, Canterbury, and private study; T. F. R. McDonnell, St. Paul's School and private study; O. Marriott, Guy's Hospital; Louisa Martindale, Royal Holloway College; Marion Bessie Mathieson, University College; H. H. Mills, Westminster Hospital, Birkbeck Institution and private study; J. Milne, Owens College; A. Moon, Guy's Hospital; R. R. Mowll, King's College; H. L. Norris, St. Thomas's Hospital; E. M. Pearse, University College, Bristol; W. E. Peck, University College; A. G. G. Plumley, Merchant Venturers' School, Bristol; E. C. Plummer, King's College; H. D. Pollard, High School and University College, Nottingham; J. H. Rhodes, St. Bartholomew's Hospital; G. M. C. Richards, High School and University College, Nottingham; H. E. Richardson, B.A., private study; A. Ricketts, University College; Adeline Mary Roberts, University Tutorial College; W. E. Ruttledge, University College; Mary E. S. Scharlieb, B.A., University College; W. P. Schreiner, Downing College, Cambridge; W. S. Sheldon, University College; W. B. Silas, University Tutorial College; H. D. Singer, Merchant Taylors' School, University Tutorial College, and private study; A. R. Spencer, Carlyon College and private study; E. Stott, Owens College; H. Stuart, University College; Margaret Helen Style, Royal Holloway College; J. G. C. Taunton, Mason College; H. J. Taylor, Owens College; H. S. Thomas, St. Bartholomew's Hospital; A. J. W. Wells, University Tutorial College; W. D. Wiggins, St. Mary's Hospital; R. S. Williams, University, Edinburgh, and University College, Liverpool; R. E. B. Wilmot, St. Mary's Hospital; B. M. Young, St. Thomas's Hospital.

### University of Aberdeen.

At a meeting of the University Court, the Marquis of Huntly presiding, the following gentlemen were appointed assistants to the professors for the winter session: Chemistry, Thomas Spencer Murray, D.Sc.; Anatomy, James Gibson, M.B.; Materia Medica, John Gordon, M.D.; Medical Jurisprudence, David Rennet, M.D. Examiners for graduation in the Faculty of Medicine were appointed as follows: Botany, R. J. Harvey Gibson, M.A., Liverpool; Zoology, J. A. Thomson, M.A., Edinburgh; Chemistry, John Hunter, F.I.C., F.C.S., Edinburgh; Physiology, George N. Stewart, M.D., Cambridge; Materia Medica, Francis Warner, M.D., London; Medical Jurisprudence, Fras. J. Allan, M.D., London; Pathology, James Lorrain Smith, M.A., M.D., Cambridge; Medicine, Sidney Coupland, M.D., London; Surgery, John C. Ogilvie Will, M.D., Aberdeen; Midwifery, G. M. Edmond, M.D., Aberdeen; Anatomy, Bertram C. A. Windle, M.D., D.Sc., Birmingham.

### The Hospital Advertiser.

### MEDICAL APPOINTMENTS—(*continued*).

**TORBAY HOSPITAL AND PROVIDENT DISPENSARY, TORQUAY.**

